# From School-Based Immunization to Family Health Centers: the Impact of a 2020 Policy Change on 13-Year-Old Tetanus-Diphtheria Vaccine Coverage in Turkey

**DOI:** 10.1007/s10728-025-00557-8

**Published:** 2026-01-23

**Authors:** Ufuk Acar, Burcu Beyazgül, Feyyaz Barlas, Harun Mesut Atmacaoğlu, İbrahim Koruk

**Affiliations:** 1 Departmant of Public Health, Harran University Faculty of Medicine, Şanlıurfa, Turkey; 2 No 14 Akabe Family Health Centre Eyyübiye, Şanlıurfa, Turkey

**Keywords:** Tetanus-Diphtheria, Family health centers, Adolescent immunization, Vaccine hesitancy, Public health policy

## Abstract

This study evaluated the impact of transferring the thirteen‑year tetanus-diphtheria booster from schools to Family Health Centers on vaccination coverage after 1 July 2020. In a two‑component descriptive cross‑sectional design conducted in the central districts of Şanlıurfa Province, annual non‑vaccination from 2018 to 2022 was tracked using administrative records; in addition, reasons for non‑vaccination were classified through face‑to‑face interviews with family physicians and on‑screen verification in nineteen Family Medicine Units selected by two‑stage stratified sampling. The primary outcome was non‑vaccination for the tetanus-diphtheria dose at age thirteen. In the research group, non‑vaccination was 23.5% in 2018, 27.2% in 2019, 22.2% in 2020 (annual total), 13.2% in 2021 and 12.3% in 2022; within 2020, rates were 32.4% before July and 11.9% after July. The absolute pre‑ versus post‑policy difference was 14.4% points and the relative risk was 0.47; the three districts likewise showed consistent declines in 2021-2022 compared with 2018-2019. Leading reasons for non‑vaccination were failure to attend after expressing intent (49.1%), outright refusal (20.9%), migration‑related mobility (13.5%), and doses administered elsewhere that were not visible in the records (8.6%). Relocating delivery to Family Health Centers strengthened access and follow‑up and was associated with improved coverage; yet intention-behaviour gaps, mobility and data fragmentation still limit attainment of the national threshold. A hybrid model combining school‑based visits with targeted completion at Family Health Centers may provide a pragmatic, low‑cost route to reach remaining groups.

## Introduction

School age is a critical period during which children’s physical, cognitive, and social development accelerates and peer interactions intensify [1]. In this stage, children not only refine mental and physical capacities but also acquire social skills and a sense of civic responsibility. Because schools bring large numbers of children together in shared settings, the risk of infectious‑disease transmission is substantial [2]. Consequently, school age is regarded as a priority for the planning and delivery of preventive health services [3].

Vaccination is among the most effective and cost‑efficient interventions for preventing infectious diseases [4]. Immunizations administered in childhood and adolescence help generate herd immunity, avert outbreaks, and markedly reduce the probability of severe clinical outcomes [5]. Beyond protection against specific infections, vaccination also lowers hospitalizations, productivity losses, and health‑care expenditures in the long term [3]. For these reasons, maintaining uninterrupted immunization programmes remains a central public‑health priority in many countries [2].

Contemporary immunization practice faces multiple challenges, ranging from logistics and workforce training to parental attitudes and economic constraints [6]. Access can be especially difficult in rural areas with limited transport; seasonal labour migration and refugee populations may also disrupt regular adherence to vaccination schedules [7]. Uneven adoption of digital record systems across regions and field‑level staffing shortfalls can further impede efforts [5]. Moreover, misinformation that spreads rapidly through mass and social media may heighten parental anxiety and undermine programme effectiveness [8].

Success in vaccination strategies requires a scientific, multi‑stakeholder approach aligned with community needs [6]. Common elements include regular training for health‑care personnel, structured collaboration between schools and primary care, deployment of mobile teams, and targeted information campaigns for parents [4]. In Turkey, following the recommendations of the National Immunization Advisory Board within the Expanded Program on Immunization, a major policy change was enacted on 3 June 2020. School‑based vaccines for grade‑1 and grade‑8 students were reassigned to the primary‑care system specifically to Family Health Centers (FHCs), within which multiple Family Medicine Units (FMUs) operate. Accordingly, from 1 July 2020 onward, the second dose of measles-mumps-rubella and the booster dose of diphtheria-tetanus-acellular pertussis-inactivated polio, previously delivered in first grade to children who had reached 48 months and were born on or after 1 July 2016, have been provided at FHCs by FMUs. As of the same date, the tetanus-diphtheria booster at age 13 is likewise administered at FHCs/FMUs [9]. Implementing the age‑13 tetanus-diphtheria dose within this new delivery model offers a timely opportunity to assess real‑world programme performance. Focusing on Province Şanlıurfa, this study examines the impact of the 2020 policy on age‑13 tetanus-diphtheria vaccination, analysing changes in coverage and the factors shaping those changes. The findings are intended to inform public‑health policy and to support the design of more effective strategies addressing vaccine refusal and vaccine hesitancy.

## Materials and methods

### Study Design, Setting, and Period

This was a two‑component descriptive cross‑sectional study conducted in the central districts of Şanlıurfa Province (Haliliye, Eyyübiye, Karaköprü). (i) Annual non‑vaccination/coverage for the tetanus-diphtheria (Td) booster at age 13 was tracked at district level for 2018-2022. (ii) Reasons for non‑vaccination were characterized through face‑to‑face physician interviews and on‑screen record verification in a cluster sample of 19 Family Medicine Units (FMUs). Policy exposure was defined as the transition on 1 July 2020 from school‑based delivery to delivery within Family Health Centers (FHCs).

### Primary Care System in Turkey and Study Context

Primary care in Turkey is delivered through FMUs that operate under FHCs. Each FMU consists of a board‑certified family physician and a family health worker (nurse/assistant), and FHCs house multiple FMUs within a shared building and infrastructure. Every FMU serves a defined population and catchment area; together, FMUs cover the entirety of a district. District Health Directorates coordinate and supervise field implementation. Before the policy change, school‑age vaccinations (including the age‑13 Td dose) were administered by District Health teams during one or two school visits; missed doses were left to families/children to complete by attending an FMU, and FMUs had no explicit proactive obligation to chase these gaps. After the change, operational responsibility shifted to FHCs/FMUs. Using the Family Medicine Information System (FMIS), FMUs receive auto‑generated “due” lists and run active recall (telephone/SMS and, where necessary, home visits). Thus, the age‑13 Td dose entered routine FMU practice and is followed within the performance‑monitoring framework. Given a performance‑based payment model in Turkish primary care, preventable failures to deliver scheduled doses may lead to pay deductions, which increases completion incentives for FMUs [10].

### Sampling Frame and Selection of Clusters

The sampling unit was the FMU serving central districts of Şanlıurfa. Within each FMU, individuals born in 2004-2009 (the target age band) constituted the cluster size. A pilot in five FMUs not included in the main sample estimated a mean cluster size of ~ 167.6 and an initial non‑vaccination proportion of ~ 13.4% for the age‑13 Td dose. To reflect clustering, a small positive intra‑cluster correlation and a corresponding design effect of ~ 2 were applied. The design objective prioritized precision rather than power: the sample was planned to estimate non‑vaccination with a two‑sided 95% confidence level and ~ ± 1.7%‑point half‑width. With the pilot mean cluster size and design effect, ~ 18 clusters were sufficient; allowing for stratified allocation (district and urban/rural) and operational contingencies, the target was rounded up to 19 clusters. A finite population correction at the cluster level was not used (conservative choice).

FMUs were selected using a two‑stage stratified approach. First, quotas were allocated to the three central districts proportional to their total FMU counts. Second, within each district, FMUs were stratified by urban/rural status and allocated proportional to stratum size. Random selection from District Health Directorate master lists yielded 19 FMUs: 8 in Haliliye, 7 in Eyyübiye, and 4 in Karaköprü.

### Data Collection and Interviews

#### Reaching Clusters

Responsible family physicians of the 19 selected FMUs were contacted by telephone, informed about the study, and scheduled for an in‑person appointment; all 19 participated.

#### Interview Procedure

Each interview lasted ~ 20 min. A brief structured form captured physicians’ sociodemographic/professional characteristics and self‑reported challenges encountered in administering the age‑13 Td booster. At the end of each interview, for 2009‑born individuals documented as unvaccinated within that FMU, the “reasons for non‑vaccination” fields were verified on‑screen in FMIS and recorded alongside researcher notes. All notes were digitized the same day.

#### Instrument and Coding

The structured form was administered face‑to‑face by the research team. Free‑text descriptions of age‑13 Td implementation challenges were recorded verbatim in the field, then grouped under thematic headings during digitization. FMIS structured reason codes and free‑text notes were reviewed and categorized separately.

#### Participation and Incentives

All 19 physicians completed the interview (19/19); no financial or material incentives were provided.

#### Recording of Vaccination Status and Administrative Data Sources

Vaccination status in this study was assessed using two complementary administrative data sources within the Turkish primary health-care system. Individual-level vaccination records and “vaccinated/unvaccinated” status were obtained from the Family Medicine Information System (FMIS). District-level annual aggregate data on eligible populations, vaccinated and unvaccinated counts for the 2018-2022 period were retrieved from the Public Health Management System (PHMS) through District Health Directorates.

The denominator used in all analyses was the number of individuals registered in these administrative systems for the relevant birth cohort and calendar year; census-based or projected district population figures were not used. Accordingly, the reported rates represent administrative coverage based on registered populations rather than true population-based coverage.

Although FMIS and PHMS constitute the routine monitoring systems of the Expanded Program on Immunization, vaccinations administered in other institutions (e.g. hospitals or Family Health Centers in different provinces) may not always be reflected in real time due to inter-institutional data transfer delays. Consequently, some individuals who were vaccinated elsewhere may temporarily appear as “unvaccinated” in administrative records, particularly in settings with high population mobility. While these systems are subject to routine monitoring and performance-based audits, individual-level record completeness was not quantitatively assessed in this study and is therefore interpreted as a potential source of conservative underestimation rather than systematic bias.

### Ethics and Permissions

Ethical Approval was granted by the Clinical Research Ethics Committee of Harran University Faculty of Medicine (07.08.2023; decision HRÜ/23.14.19). Institutional permission to use individual‑level records was obtained from the Şanlıurfa Provincial Health Directorate. Participation was voluntary and verbal informed consent was obtained from physicians. All procedures complied with institutional standards and the Declaration of Helsinki.

### Statistical Analysis

Analyses were performed in IBM SPSS Statistics 26.0 (IBM Corp., Armonk, NY, USA). Categorical variables are summarized as n (%); continuous variables as mean ± SD. Between‑year differences were tested using a Pearson χ² on the 2 × 5 table. Linear trend was assessed by Linear‑by‑Linear Association (Cochran-Armitage trend test) and, additionally, by logistic regression with year as an ordinal predictor to report the per‑year odds ratio (OR). Pre‑specified contrasts pre‑ vs. post‑policy and within‑2020 (pre‑ vs. post‑July 1) were evaluated by two‑proportion z‑tests; absolute difference in percentage points (pp), risk ratio (RR), and 95% confidence intervals (95% CI) were reported. All tests were two‑sided (α = 0.05). Because annual values are administrative totals, inferences were interpreted as descriptive rather than model‑based.

## Results

Among the 19 participating family physicians, 84.2% were male; the mean age was 34.7 ± 6.8 years and the mean length of practice was 8.9 ± 5.8 years.

In the research group, annual non‑vaccination for the age‑13 tetanus-diphtheria (Td) booster was 23.5% (307/1305) in 2018, 27.2% (344/1263) in 2019, 22.2% (325/1467; annual total) in 2020, 13.2% (175/1324) in 2021, and 12.3% (163/1326) in 2022 (Table 1, Panel A). Between‑year heterogeneity was significant (Pearson χ²(4) = 144.4; *p* < 0.001), and the linear trend indicated a year‑on‑year decrease (OR per year = 0.79; 95% CI 0.76-0.83; *p* < 0.001).

**Table 1:** Annual 13-year Td non-vaccination rates and statistical comparisons (Research group, 19 FMUs)

Panel A — Annual distribution						
	**Year / Contrast**	**Eligible (*n*)**		**Unvaccinated *n* (%)**		
	2018	1305		307 (23.5%)		
*Pre-policy change*	2019	1263		344 (27.2%)		
	2020 (pre-July 1)	734		238 (32.4%)		
	2020 (post-July 1)	733		87 (11.9%)		
	2020 (total)*	1467		325 (22.2%)		
*Post-policy change*	2021	1324		175 (13.2%)		
	2022	1326		163 (12.3%)		

Panel B — Summary comparisons						
			**Effect measure**		**Result**	
**Between-year heterogeneity** (2018, 2019, 2020 total, 2021, 2022)			χ² (df = 4)		144.4; *p* < 0.001	
**Linear trend **(2018→2022)			OR (per-year)		0.79 (95% CI 0.76-0.83); *p* < 0.001	
**Pre- vs. post-policy**			Difference (pp)		14.4 (95% CI 12.5-16.2); *p* < 0.001	
**Pre- vs. post-policy**			RR		0.47 (95% CI 0.42-0.52); *p* < 0.001	
**2020** (pre- vs. post-July 1)			Difference (pp)		20.6 (95% CI 16.4-24.7); *p* < 0.001	
**2020** (pre- vs. post-July 1)			RR		0.37 (95% CI 0.29-0.46); *p* < 0.001	

For trend analysis, 2020 values were combined as “2020 (total)”

All tests are two-sided (α = 0.05). Given record-level counts, inferences are descriptive

*FMU* Family Medicine Unit, *RR* risk ratio, *CI* confidence interval, *pp* percentage points

Before the policy change (2018-2019 and 2020 pre‑July 1), the non‑vaccination rate was 26.9% (889/3302); after the change (2020 post‑July 1, 2021-2022), it was 12.6% (425/3383). The absolute difference was 14.4% points (95% CI 12.5-16.2; *p* < 0.001) and the risk ratio (RR) was 0.47 (95% CI 0.42-0.52; *p* < 0.001). Within 2020, non‑vaccination was 32.4% (238/734) pre‑July 1 vs. 11.9% (87/733) post‑July 1, an absolute difference of 20.6% points (95% CI 16.4-24.7; *p* < 0.001) with RR = 0.37 (95% CI 0.29-0.46; *p* < 0.001) (Table 1, Panel B). Note: For trend testing, 2020 values were combined as “2020 (total).”

Of the 19/19 physicians interviewed, 13 (68.4%) reported at least one challenge in administering the age‑13 Td booster. Among those reporting challenges, the most frequent issues were difficulties reaching families and/or children (38.5%), belief that vaccination is unnecessary (23.1%), refusing to attend the Family Health Center (FHC) for vaccination (15.4%), outright refusal of vaccination (15.4%), and inability to integrate doses administered outside the FHC (e.g., hospital) into the information system, with implications for performance‑based pay (7.7%). Percentages are calculated among physicians who reported at least one challenge (*n* = 13) (Table 2).

**Table 2:** Problems/challenges faced by family physicians during vaccination practices (*n*=13)

Problems/Challenges	*n*	%
Difficulties in reaching families and/or children	5	38.5%
Families adopting the belief that vaccination is unnecessary	3	23.1%
Refusal to visit the Family Health Center for vaccination	2	15.4%
Refusal of vaccination	2	15.4%
Inability to integrate vaccines administered outside the FHC (e.g., hospital) into the system, resulting in performance-based pay cuts	1	7.7%

Using Family Medicine Information System (FMIS/AHBS) records verified during interviews, the leading reasons for non‑vaccination among the 2009‑born subgroup (*n* = 163) were “promised to get vaccinated but did not show up” (49.1%; 80/163), vaccine refusal (20.9%; 34/163), migration‑related mobility (13.5%; 22/163), no record due to new registration or vaccination elsewhere (8.6%; 14/163), fear of injection (3.7%), supply issues (3.1%), and postponement due to allergy or illness (1.2%) (Table 3).

**Table 3:** Distribution of reasons for not receiving the Td vaccine according to FMIS in the research group (*n*=163)

Reasons for Non-Vaccination	*n*	%
Promised to get vaccinated but did not show up	80	49.1
Refusal of vaccination	34	20.9
*Migration	22	13.5
No record due to new registration or vaccine administered elsewhere	14	8.6
Child’s fear of injection	6	3.7
Supply issue	5	3.1
Did not vaccinate due to allergy or illness	2	1.2
Total	163	100

*Seasonal agricultural workers and refugees were identified as the primary causes of migration

*FMIS* family medicine information system

At district level (Haliliye, Eyyübiye, Karaköprü), non‑vaccination increased from 2018 to a peak in 2020 and then declined markedly in 2021-2022 (e.g., 2018→2020→2022: Haliliye:28.7%→35.7%→22.3%; Eyyübiye:33.6%→37.1%→28.4%; Karaköprü:22.4%→29.4%→16.9%). Year‑to‑year differences were significant for all districts (Haliliye χ²(4) = 562.3; Eyyübiye χ²(4) = 272.8; Karaköprü χ²(4) = 369.1; all *p* < 0.001), with decreasing linear trends (OR per year: Haliliye 0.90 [95% CI 0.88-0.91]; Eyyübiye 0.92 [0.91-0.94]; Karaköprü 0.88 [0.86-0.90]; all *p* < 0.001). Summary pre‑ vs. post‑policy contrasts (excluding 2020 as a transition year) also showed significant absolute differences and risk ratios consistent with improvement (Table 4).

**Table 4:** Annual 13-year Td non-vaccination rates and statistical comparisons (for Haliliye, Eyyübiye, Karaköprü districts)

Panel A — Annual distribution						
		**Year / Contrast**		**Eligible (*n*)**		**Unvaccinated *n* (%)**
**Haliliye**		2018		8412		2414 (28.7%)
		2019		8538		2807 (32.9%)
*Policy transition year*		2020 (total)*		8628		3084 (35.7%)
		2021		8779		2086 (23.8%)
		2022		8869		1982 (22.3%)
**Eyyübiye**		2018		9629		3237 (33.6%)
		2019		9648		3447 (35.7%)
*Policy transition year*		2020 (total)*		8502		3157 (37.1%)
		2021		8633		2427 (28.1%)
		2022		8541		2429 (28.4%)
**Karaköprü**		2018		4024		901 (22.4%)
		2019		4523		1264 (27.9%)
*Policy transition year*		2020 (total)*		4880		1435 (29.4%)
		2021		5189		928 (17.9%)
		2022		5454		923 (16.9%)

Panel B — Summary comparisons						
			**Effect measure**		**Result**	
**Haliliye**						
Between‑year heterogeneity (2018-2022)			χ² (df=4)		562.3; *p*<0.001	
Linear trend (2018→2022)			OR (per‑year)		0.90 (95% CI 0.88-0.91); *p*<0.001	
Pre‑ vs. post‑policy			Difference (pp)		7.8 (95% CI 6.8-8.7); *p*<0.001	
Pre‑ vs. post‑policy			RR		0.75 (95% CI 0.72-0.78); *p*<0.001	
**Eyyübiye**						
Between‑year heterogeneity (2018-2022)			χ² (df=4)		272.8; *p*<0.001	
Linear trend (2018→2022)			OR (per‑year)		0.92 (95% CI 0.91-0.94); *p*<0.001	
Pre‑ vs. post‑policy			Difference (pp)		6.4 (95% CI 5.4-7.3); *p*<0.001	
Pre‑ vs. post‑policy			RR		0.82 (95% CI 0.79-0.84); *p*<0.001	
**Karaköprü**						
Between‑year heterogeneity (2018-2022)			χ² (df=4)		369.1; *p*<0.001	
Linear trend (2018→2022)			OR (per‑year)		0.88 (95% CI 0.86-0.90); *p*<0.001	
Pre‑ vs. post‑policy			Difference (pp)		7.9 (95% CI 6.8-9.1); *p*<0.001	
Pre‑ vs. post‑policy			RR		0.69 (95% CI 0.65-0.73); *p*<0.001	

For districts, pre‑ vs. post‑policy comparisons pool 2018-2019 vs. 2021-2022 (2020 treated as transition and excluded). For the research group, pre‑ vs. post‑policy uses the within‑2020 split (pre/post‑July 1) plus 2018-2019 and 2021-2022, respectively. All tests are two‑sided (α=0.05); given record‑level counts, inferences are descriptive

*RR* risk ratio, *CI* confidence interval, *pp* percentage points

**Fig. 1:**
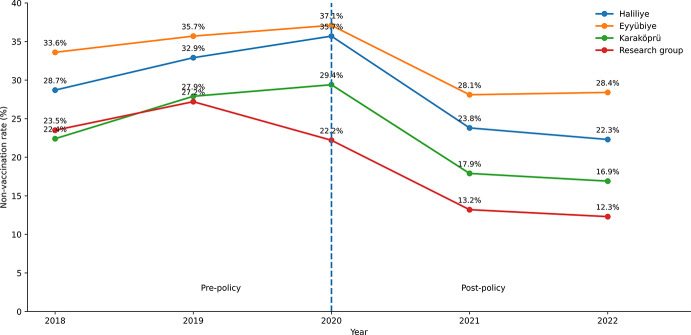
Annual frequency of Td non-vaccination in the central districts and in the research group

Figure 1 visually depicts the pre‑ and post‑policy periods separated by the 2020 transition line, showing the temporal alignment of declines in both the districts and the research group; the decline is steeper in the research group (2018 23.5%, 2019 27.2%, 2020 22.2% total, 2021 13.2%, 2022 12.3%), consistent with the formal tests reported above.

## Discussion

This study shows a pattern consistent with a substantive reduction in non‑vaccination following the transfer of the age‑13 tetanus-diphtheria (Td) booster from school‑based delivery to Family Health Centers (FHCs). In the research group, both between‑year heterogeneity and the linear trend analyses indicated significant decreases, and the within‑2020 half‑year contrast aligned temporally with the policy break. At district level, declines observed in 2021-2022 relative to 2018-2019 were similarly consistent. Taken together, and particularly given the magnitude of the within‑2020 drop in the same clusters, these changes are unlikely to be explained by baseline differences alone and suggest that post‑policy processes accelerated access and completion.

Baseline non‑vaccination in the research group was lower than in other central districts. Plausible explanations include comparatively better record integrity in some Family Medicine Units (FMUs) for example, prompt entry of doses administered in hospitals or private settings differences in urban-rural composition and migration dynamics, and the presence of FMU‑level practices (active recall, flexible appointment windows) that had already been adopted before the policy change. Because clusters were selected by proportional stratified random sampling with full participation (19/19), selection bias was mitigated, although cluster heterogeneity cannot be eliminated; interpretation therefore emphasizes the time‑trajectory within the same clusters.

School‑ and FHC/FMU‑based strategies are complementary rather than mutually exclusive. School delivery offers on‑site access and the ability to reach large cohorts in one visit, whereas FMUs provide individualized follow‑up, routine recall, and flexible scheduling that support targeted completion [11-13]. Balancing these mechanisms to local context appears crucial for closing the “intention-behaviour gap” in this age group, especially where school and workday schedules conflict or parental accompaniment is needed; the behavioural and immunization literature highlights the value of active recall, suitable time windows, and focused communication in converting intention into uptake [14, 15]. Consistent with this, the present study identified “promised to get vaccinated but did not show up” as the most frequent reason for non‑vaccination, underscoring the operational challenges of relying solely on FMU attendance.

Turkey’s primary care has long incorporated performance‑based elements based on government programmes. Bringing the age‑13 Td dose under FMU responsibility in 2020 integrated it into the routine recall/monitoring chain at FMUs rather than introducing an entirely new incentive structure; the findings are compatible with this institutional framing, although the study design does not estimate causal effects and the relative contribution of incentives is discussed as interpretation rather than as a quantified impact [10].

Under the Expanded Program on Immunization, the national coverage target for school‑age antigens is ≥ 95%. In the research group, post‑policy coverage approached but did not reach that threshold (≈ 87-88%), indicating a residual operational gap amenable to pragmatic solutions [9]. The prominence of “no‑show after intent” points to the need for multichannel reminders and convenient catch‑up options; vaccine hesitancy/refusal (20.9%) reflects broader issues of trust, information environments, and social norms, for which transparent communication, parent‑centred counselling, and trust‑building measures are essential [15, 16]. Migration‑related mobility (13.5%) creates coverage gaps; school‑based catch‑up days, mobile teams, and targeted transport can improve access for these populations [17]. Differences in record integrity (8.6%) notably delays in inter‑facility data transfer may underestimate coverage; real‑time data integration and bilateral verification among providers are preconditions for accurate monitoring and fair performance accounting [18, 19].

Overall, the decline in non‑vaccination in the research group does not appear to arise from a single mechanism, but from the joint management of access (school), follow‑up (FMU), data quality, and behavioural factors. Operating school‑based sessions together with targeted completion at FMUs is consistent with the literature and aligns with facilitators and barriers identified in the present fieldwork [11-15, 19].

## Strengths and Limitations

This analysis is based on administrative totals and is therefore descriptive; individual‑level causal effects were not estimated. Although clusters were chosen by proportional stratified random sampling with full participation, residual baseline heterogeneity is possible; inference focuses on changes over time within the same clusters. Inter‑institutional transfer delays can leave vaccinated individuals recorded as “unvaccinated,” biasing coverage downward. Reasons for non‑vaccination were compiled for unvaccinated individuals born in 2009 and may not generalize to other birth cohorts. Facilitators abstracted from physicians’ free‑text notes are qualitative and were not quantified. The post‑policy window partially overlaps with the COVID‑19 period; concurrent local initiatives or contextual factors (school attendance, mobility) cannot be fully excluded.

## Conclusions and Recommendations

Transitioning delivery of the age‑13 Td booster from schools to FHCs was associated with a meaningful improvement in coverage, yet achieving the ≥ 95% national threshold will require closing operational gaps. The most actionable path is to combine the complementary strengths of schools and FMUs: standardize active recall, flexible hours, and short catch‑up sessions; ensure real‑time integration and bilateral verification of doses administered in hospitals or private facilities; provide mobile/on‑site solutions for migration‑affected or hard‑to‑reach groups; and strengthen team workflows and school-FMU micro‑collaboration. Our central policy recommendation is: “A hybrid model composed of school‑based mass visits plus targeted completion at FMUs can reach the remaining small groups with lower cost, fewer personnel, and simpler logistics, while combining the FMU’s capacity for individualized follow‑up with the school’s on‑site access. This pragmatic framework offers a credible route toward the ≥ 95% target.”

## Data Availability

The data that support the findings of this study are available from Şanlıurfa Provincial Health Directorate. Restrictions apply to the availability of these data, which were used under license for this study. Data are available from the authors with the permission of Şanlıurfa Provincial Health Directorate.
